# The impact of puff frequency on respirable particulate matter in mainstream cigarette smoke

**DOI:** 10.1111/crj.13592

**Published:** 2023-01-31

**Authors:** Alfayo K. Maiyo, Benjamin K. Korir, Joshua K. Kibet

**Affiliations:** ^1^ Department of Chemistry and Biochemistry Moi University Eldoret Kenya; ^2^ Department of Chemistry Egerton University Njoro Kenya

**Keywords:** biochar, biological damage, inhalation, particulate matter, PM_2.5_, puff time, tobacco

## Abstract

**Background:**

Inhalation of particulate matter (PM) from cigarette smoke is hazardous to smokers and non‐smokers. This contribution simulates the deposition of cigarette PM on the lung surface by trapping tobacco smoke particulates on *Croton megalocarpus* biochar. This study investigated one commercial cigarette (MM) and one local cigarette (RR).

**Methodology:**

Biochar was incorporated into the filters of MM and RR cigarettes in order to adsorb PM from mainstream cigarette smoke. A weighed 5 mg of biochar with adsorbed cigarette PM was analyzed using a scanning electron microscope and a Fourier transform infrared spectrometer. The size distribution of cigarette smoke particulates was processed using ImageJ software.

**Results:**

At 15 s puff time, the mean particulate diameters for the commercial and the local cigarettes, respectively, can be classified as coarse ≈ PM_10_. Conversely, the mean particulate diameter at 2 s puff time for the commercial cigarette falls under the ultrafine classification of ≤PM_2.5_, whereas at the same puff time, the mean particulate diameter for the local cigarette was approximately PM_2.5_. Data from Fourier transform infrared spectroscopy indicate the PM in the two model cigarettes contains aromatic structures that feature the C=C bond characterized by an intense absorption band at δs (1600 cm^−1^).

**Conclusions:**

This study found that PM in mainstream cigarette smoke depends on puff time. Although cigarette smoking was conducted for two model cigarettes, this study can be extended to any other form of cigarette. Moreover, this study emphasizes the need for comprehensive studies on real‐world cigarette smoking conditions, taking into account cigarette smokers who use larger puff volumes.

## Graphical abstract

Tobacco smoke is a toxic gas‐phase cocktail consisting of a broad range of particulate matter (PM) comprising organics, metals, and free radical intermediates. Airborne PM is one important aspect of grave health concerns. Fine particles with an aerodynamic diameter of less than 2.5 μm are considered fatal because they can penetrate deeper areas of the human respiratory airway and even the blood circulatory system.

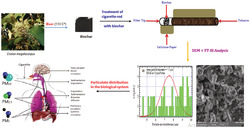

## 1 BACKGROUND

Statistics by the World Health Organization (WHO) estimate that there are approximately 1.1 billion smokers globally, with this figure remaining unchanged since 2000, and represent 20% of the global adult population.^1^ During cigarette smoking (CS), two pathways of smoke generation dominate—mainstream cigarette smoke (MCS), which emerges from the butt end, and side stream cigarette smoke, which is emitted into the surrounding air from the burning end of the cigarette.^2^ Particulate matter (PM) is dependent on size, shape, and solubility and has the potential to cause harm.^2^, ^3^ The smaller the particles are, the deeper they can be inhaled, whereas coarse particles generally pass the nose and throat before entering the lungs.^3^ Fine particles penetrate the gas exchange regions of the lung, and some may even cross into the bloodstream.^4^ Some elements in tobacco complex mixture consist of volatile or semi‐volatile compounds, and cigarette smoke has been identified as environmental endocrine disrupting chemicals (EDCs) and precursors for grave health ailments including cancer, oxidative stress, and coronary diseases.^5^, ^6^ Ideally, cigarette smokers deposit up to 20 mg of tar in the lungs per cigarette smoked, which is about 1 g of tar per day, depending on the cigarettes smoked by the individual.^7^

The motivation behind this study is to investigate cigarette smoke particulate emissions from two cigarettes coded MM and RR at different puff times, the results of which can be extrapolated to other types of cigarettes. PM has been considered a serious health concern, and it is attracting significant research interest in combating the tobacco epidemic.^8^, ^9^ Respirable PM has been categorized into three major sizes—PM_10_ (coarse) and PM_2.5_ (fine), with extended subdivision to ultrafine PM_1_.^10^ Ultrafine particulates are considered extremely severe to the biological system as they can penetrate deep into the respiratory landscape and blood vessels.^11^ This study investigates PM emitted in MCS from two types of cigarettes, MM and RR, using the state‐of‐the‐art size distribution software packages, ImageJ and Igor (ver. 5.0).

Tobacco burning undergoes various mechanistic processes such as coagulation, hygroscopic growth, condensation and evaporation, changes in composition, and changes in inhalation characteristics to form numerous sizes of particulates that are small enough to be deposited in the respiratory airway of the smoker, resulting in exposure of harmful toxicants that can precipitate cell injury and disease.^12^ It is estimated that 10^12^ particles per cigarette are released in MCS.^13^ This high concentration of particulates per cigarette is detrimental to human health. Furthermore, tar‐bound free radicals (contain ˃10^17^ free radicals per gram) are long‐lived and have long lifetimes when compared with gas‐phase bound free radicals, which are short‐lived (contain ˃10^17^ free radicals per puff).^7^ PM in cigarette smoke are precursor for lung cancer, chronic obstructive pulmonary disease (COPD), and cardiovascular diseases, implying that the etiological risks of MCS are mainly caused by inhalation of PM.

Smoking is the main precursor for COPD, an inflammatory disorder characterized by a progressive and largely irreversible respiratory tract obstruction; therefore, a greater understanding of cellular and molecular mechanisms that contribute to the pathogenesis of COPD is very important given the extremely addictive nature and chronic persistence of CS.^14^ One way of classifying PM is by defining the deepness of penetration into the respiratory airway.^15^ Accordingly, the smaller the particles, the deeper they penetrate the lung microphages and the bloodstream, causing severe etiological risks.^16^ Furthermore, smaller particles have a higher ability to adsorb toxic organic molecules and inorganic metal particulates that can penetrate through the blood and the nervous system into the brain matter and various organs.^17^, ^18^ There is an inverse relationship between particle size and associated health risks. However, particulates containing metal particulates cause severe epidemiological consequences such as induced pulmonary macrophages and dendritic cells, which excite the release of pro‐inflammatory cytokines and cell sensitization.^13^

## 2 METHODS

### 2.1 Sample preparation

**FIGURE 1:**
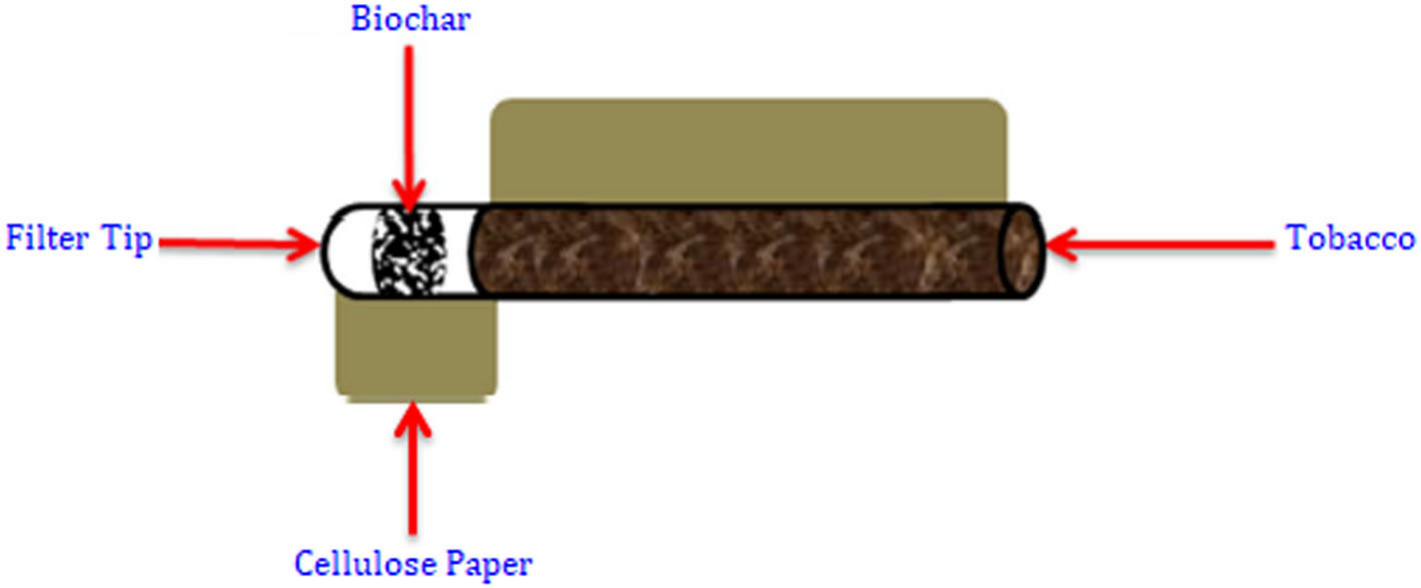
Cigarette rod treated with *C. megalocarpus* seed husk biochar. Adapted from Maiyo et al.^19^

In this study, scanning electron microscopy (SEM) and Fourier transform infrared (FTIR) spectroscopy analytical techniques were used to analyze cigarette particulate. Cigarettes coded MM and RR, respectively, were conditioned under ambient conditions. Approximately 0.24 g of *C. megalocarpus* biochar was incorporated into the filter of the cigarettes under investigation, as shown in Figure 1. Specially designed smoking apparatus that simulate CS was used following the procedure of Maiyo et al.^19^

### 2.2 Smoking procedure

The cigarettes used in this study were smoked based on a procedure representative of CS—35 mL puff volume with 2 s residence time every 60 s.^20^ During smoking, the contact time varied from 2 to 15 s to simulate the puffing regimes exhibited by various cigarette smokers. The detailed procedure is reported in our previous study.^19^

### 2.3 FTIR spectroscopy

FTIR spectroscopy is remarkably one of the most important and versatile analytical techniques available for the analysis of surface functionalities of PM. Absorption spectra were collected according to the procedure developed in the literature.^21^, ^22^ Attenuated total reflection FTIR spectra were taken following the procedure of Mili et al.^21^ The sample size used for FTIR was ≈5 mg.

### 2.4 SEM and image processing

Approximately 5 mg of *C. megalocarpus* seed husks biochar with particulate deposition of toxic molecular products adhered to aluminum SEM stubs following the procedure of Maiyo et al.^19^ ImageJ software (National Institute of Health [NIH], Bethesda, MD) and Igor ver. 5.0 (Wavemetrics, Lake Oswego, OR, USA) graphing software programs were used to estimate the mean aerodynamic diameter of cigarette smoke. An average of 100 particulates were measured from SEM micrographs. ImageJ software is a Java‐based public domain image processing and analysis program that is freely available and open source and developed at the NIH, USA.^23^

## 3 RESULTS

**FIGURE 2:**
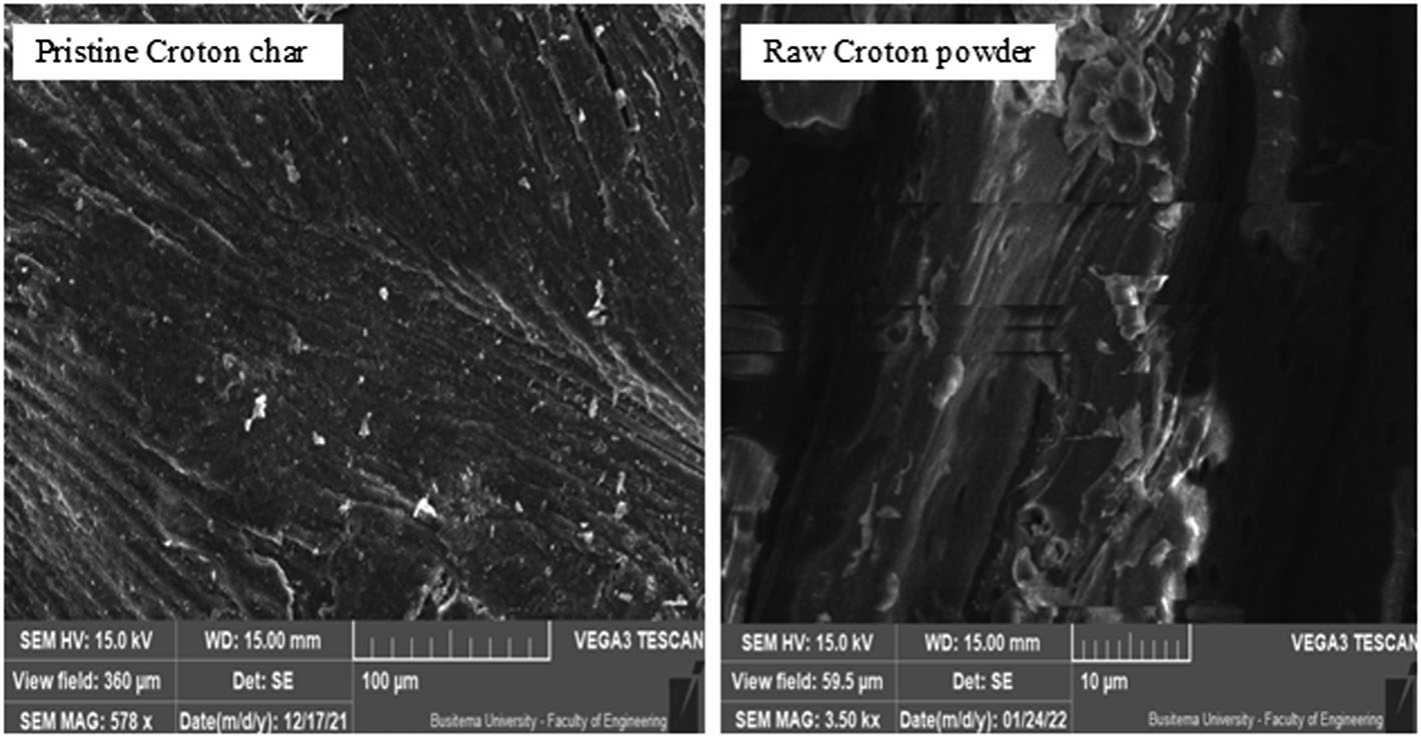
Micrographs of pristine *Croton* seed husk biochar and raw *Croton* husk powder at an associated magnification ×800 at 100 and 10 μm, respectively.

**FIGURE 3:**
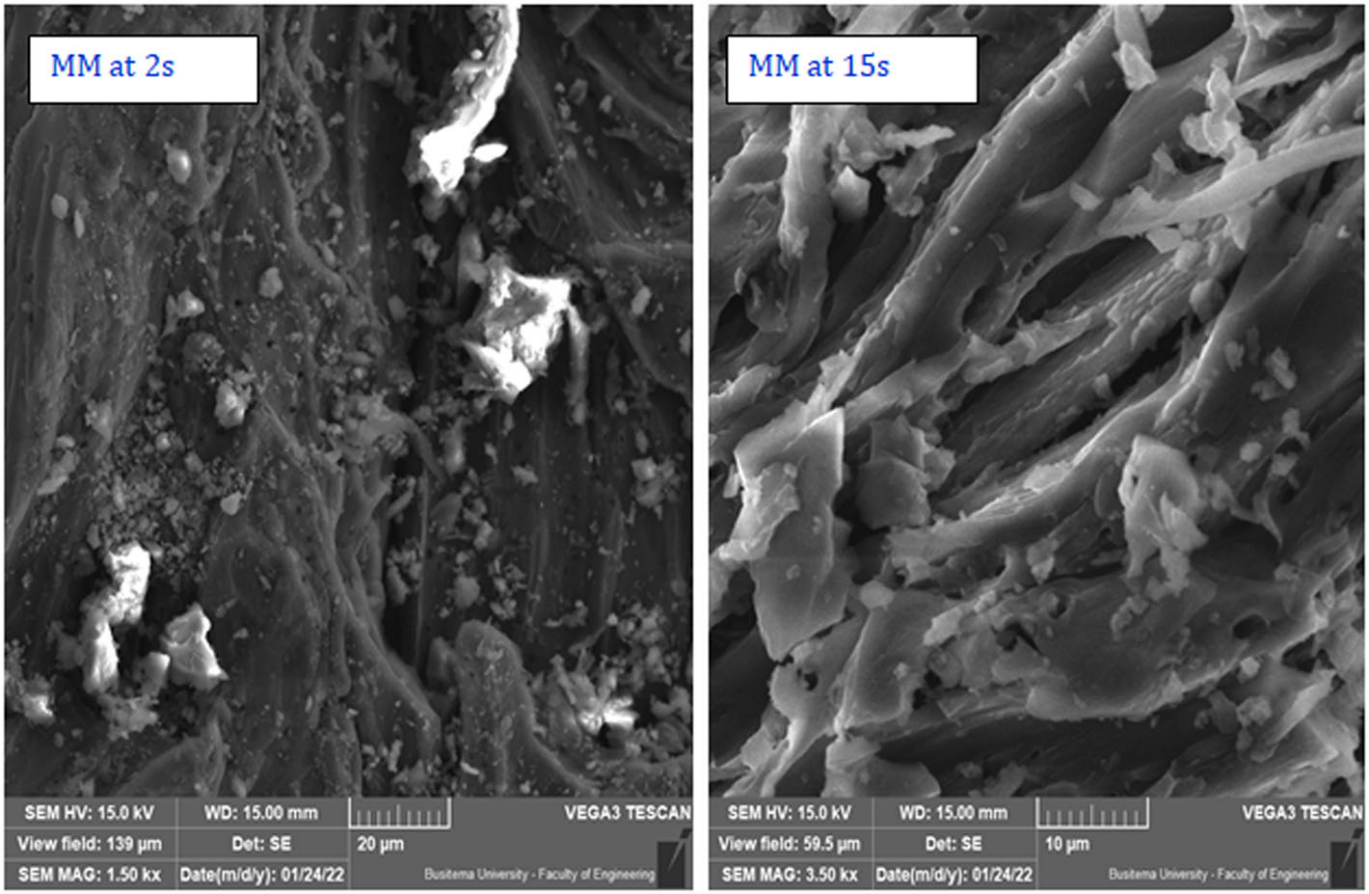
Particulate depositions of cigarette particulates from MM cigarette on biochar at 2 and 15 s puff time at an associated magnification of ×800 at 20 and 10 μm, respectively.

**FIGURE 4:**
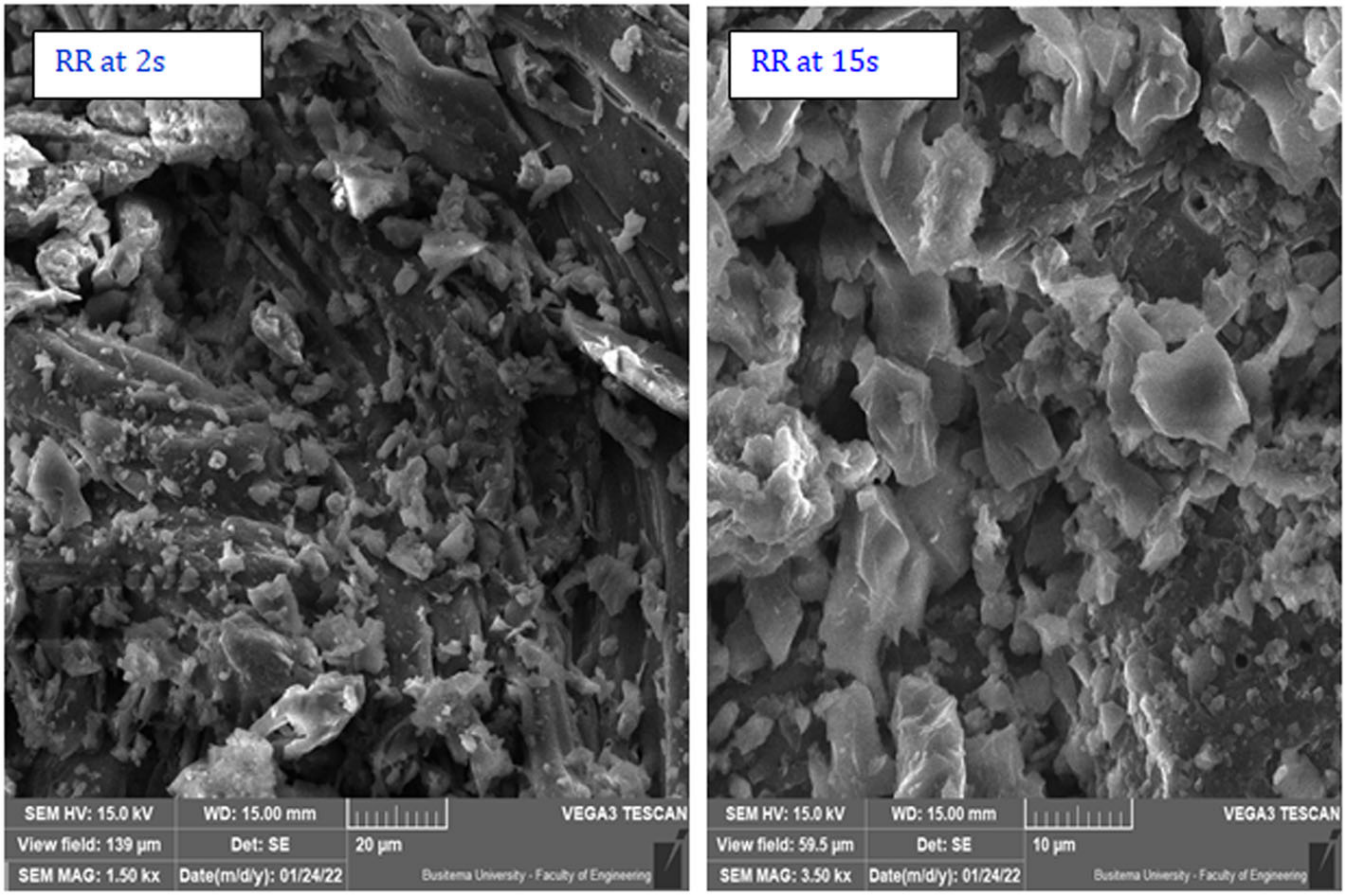
Particulate depositions of cigarette particulates from RR cigarette on biochar at 2 and 15 s puff time at an associated magnification of ×800 at 20 and 10 μm, respectively.

Scanning electron micrographs of *C. megalocarpus* seed husks biochar, reported in Figure 2, was obtained at respective scales of 100 and 10 μm. Also, Figure 2 gives the surface topography of pristine *Croton* char and that of untreated *Croton* raw powder. In these micrographs, no PM from cigarette smoke was exposed because they are the controls in the study. The morphology of biochar treated with MM and RR cigarettes, according to Figures 3 and 4, shows particulate deposition of cigarette particulates taken at an associated magnification of ×800 at residence times of 2 and 15 s.

**FIGURE 5:**
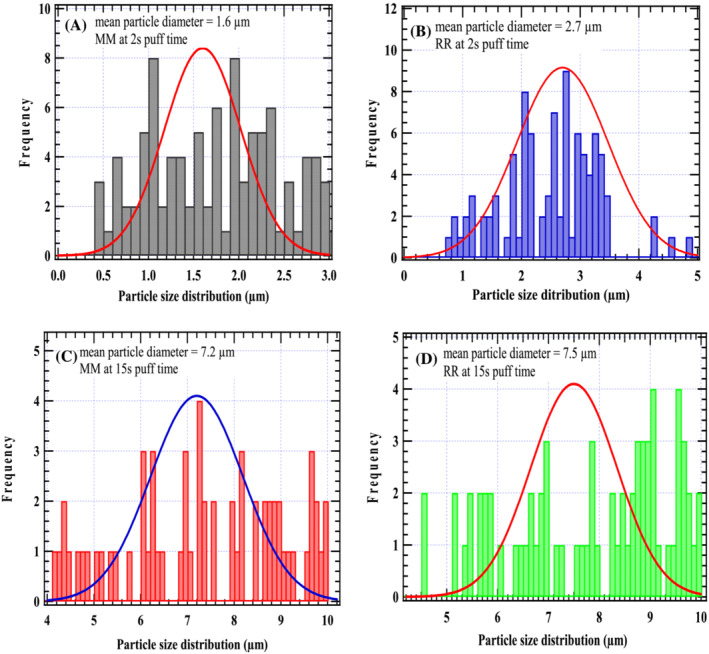
Average diameter of cigarette particulate matter from MM (A and C) and RR (B and D) cigarettes at residence times of 2 and 15 s.

Smoke particulates deposited on *C. megalocarpus* biochar from the MM and RR cigarettes at puff times of 5 and 10 s are presented in Table 1. The mean particle size diameter of particulates from the treated MM cigarette was 3.2 and 4.6 μm at puff times of 5 and 10 s, respectively, whereas for the treated RR cigarette, the mean particulate size was found to be 4.3 and 6.4 μm, correspondingly. It is clear from Figure 5 and Table 1 that as the puff time increases, the particulate size of cigarette smoke deposited on biochar increases significantly. The deposition of cigarette particulate size for RR cigarettes is higher than the particulate size deposition from MM cigarettes.

**TABLE 1:** Mean particulate diameters measured for commercial and local cigarettes at specific residence times

Type of cigarette	Puff residence time (s)	Mean particulate diameter (μm)
Standard cigarette (MM)	5.0	3.2
	10.0	4.6
Local cigarette (RR)	5.0	4.3
	10.0	6.4

**FIGURE 6:**
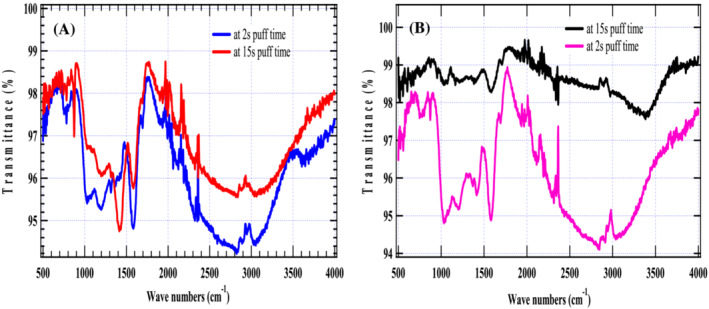
Fourier transform infrared overlay spectra for particulate matter from (A**)** MM cigarette and (B) RR cigarette at 2 and 15 s residence times.

Figure 5 reports that the mean particulate size deposited on biochar from the MM cigarette was found to be 1.6 and 7.2 μm at a residence time of 2 and 15 s, respectively. On the other hand, the mean particulate size from the RR cigarette at the same residence times was 2.7 and 7.5 μm, respectively. Figure 6 gives similar FTIR absorption spectra for the two model cigarettes implying that the surface functionalities of the PM are the same. Nonetheless, weaker absorption bands are observed for the local cigarette RR compared with the absorption bands for the standard cigarette MM. It is also observed that the absorption bands decrease significantly as the puff time increases from 2 to 15 s. This can be attributed to the effect of free radical recombination, bond formation, and polymerization as the puff time is increased.

## 4 DISCUSSION

### 4.1 PM characterization in MCS

The size distribution of cigarette smoke particulates is an essential factor in predicting the deposition fraction of the inhaled particles in various regions of the respiratory tract, as reported in earlier studies by Sahu et al.^13^ It is evident from this study that at longer puff times, for instance at 15 s puff time, the mean particulate diameters for the MM and the RR cigarettes, respectively, can be classified as coarse ≈ ${\mathrm{PM}}_{10}$. On the other hand, the mean particulate diameter at 2 s puff time for the standard cigarette falls under the ultrafine classification of $\leq {\mathrm{PM}}_{2.5}$, whereas at the same puff time, the mean particulate diameter for the local cigarette was approximately ${\mathrm{PM}}_{2.5}\;\left(2.7\mathrm{\mu m}\right)$ that falls under the fine PM category. Therefore, as the smoke ages from 2 to 15 s, there is significant coagulation, accumulation, and particle growth resulting from an increase in the diameter of particulates on the surface of biochar. At a puff time of 15 s, the particulate size increases by 4.5 and ≈3 times the particulate size at 2 s puff time for the standard cigarette and the local cigarette, respectively. This observation is very important because, at longer residence times, cigarette smokers can avoid the serious health consequences of inhaling fine and ultrafine particulates at shorter residence times.

The FTIR spectrum of cigarette smoke PM was shown in Figure 6. The spectral peak at1600 cm^−1^ is attributed to a C=C double bond characteristic of an aromatic structure in cigarette smoke particulates, whereas the absorption peaks, 2800 and 2900 cm^−1^, represent the asymmetrical and symmetrical stretching of –CH_2_– functionality for long‐chain aliphatic hydrocarbons. Besides, moderately intense absorption bands at 1400 cm^−1^ are signatures for –CH_2_ bending modes in aromatic structures. Similarly, the peak at 1200 cm^−1^ shows the presence of –O–CR (ether) groups. The absorption bands at 850 and 800 cm^−1^ confirm the presence of –CH_2_ on the surface of biochar.

From the results herein presented, the average diameter size was high for both MM and RR cigarettes at a puff time of 15 s (7.2 and 7.5 μm) when compared with 1.6 and 2.7 μm mean particulate size at a puff time of 2 s. This indicates that, as the smoke ages, that is, from 2 to 15 s residence times, there is a significant coagulation, accumulation, and particle growth, resulting in increased diameter of smoke PM.^24^ In reality, this process takes place in the trachea once the smoker fills and holds fresh mainstream smoke in the mouth before inhaling it into the respiratory tract.^13^, ^25^ Although this process is affected by the evaporation of volatile constituents and high dilution, it correlates very well with previous data in combustion systems.^21^, ^24^

### 4.2 Pathophysiological considerations of exposure to PM

**FIGURE 7:**
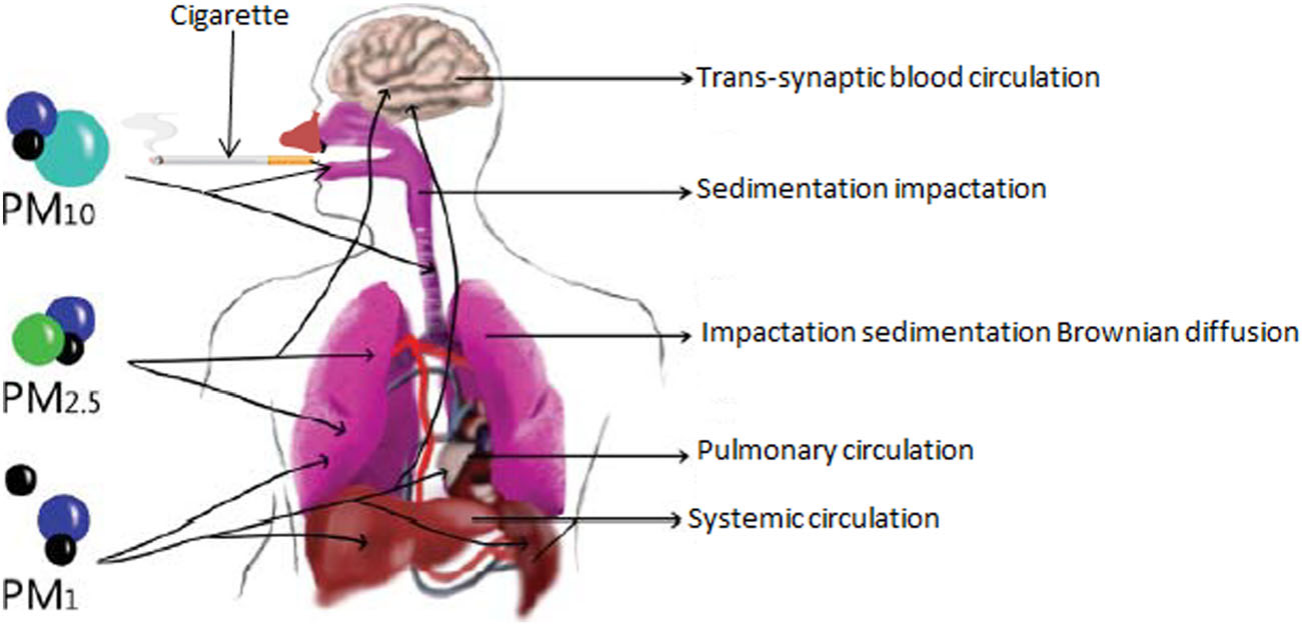
Cigarette particulate size distribution in the respiratory landscape. Modified from Broday and Robinson.^25^

CS induces oxidative stress resulting in chronic inflammation and recruitment of inflammatory cells to the airways by activation of epithelial cells, neutrophils, and lymphocytes.^7^, ^26^ Furthermore, CS increases the severity of disease‐causing agents and promotes the risk of pulmonary infections.^27^ The fine and ultrafine PM reported in this work are potent because they can be inhaled deeper into the respiratory landscape, as earlier reported by Kwon et al.^28^ Particulate emissions having an aerodynamic diameter ≤2.5 μm can cross the biological respiratory filters and are precursors for alveoli injury and irreversible damage to the lung microphages.^28^, ^29^ Moreover, ultrafine PM initiates upper and lower respiratory inflammation, malignant lung growth, rib cage malfunctions, and, ultimately, cancer of the lungs.^29^, ^30^ Evidently, fine and ultrafine PM exposure from ambient air pollution and cigarette smoke has been associated with high risks of cardiovascular death and other coronary complications, including cancer and bronchitis.^31^ Therefore, in order to protect cigarette smokers and non‐smokers, it should be mandatory for tobacco companies and importers of tobacco products to declare the PM emissions in mainstream and second‐hand smoke of all tobacco products to the government and public authorities.^32^ Figure 7 presents a summary of the particulate size distribution in the respiratory airway and their etiological impact on other biological organs.^25^

Respirable cigarette smoke particulates with an aerodynamic diameter of 2.5–10 μm and fine particles smaller than 2.5 μm aerodynamic diameter are considered toxic and pose serious etiological risks.^33^ Fine PM of less than 2.5 μm in size are detrimental to the human biological system, especially because they can penetrate very deep areas of the human respiratory airways and can permeate through the blood circulatory system.^34^ Cigarette smoke particulates containing toxic metals and organics are the major cause of oxidative stress among cigarette smokers.^30^ The colonization of bacteria noted in the lower airway of COPD patients is reported to increase the translocation of *Streptococcus pneumoniae* into the lung during cigarette smoking, promotes inflammation, and is very important in the overactivation of immune cells of the upper respiratory airway and the lung.^35^ The exact toxic components of cigarette smoke and the mechanisms involved in CS‐related cardiovascular dysfunction are largely unknown, but CS increases inflammation, thrombosis, and oxidation of low‐density lipoprotein cholesterol.^29^ Although the current tobacco reform policy emphasizes abstinence, the risk of coronary morbidity and mortality in patients with a smoking history is still significantly high.^36^ Ultimately, CS exposes the individual to numerous clinical atherosclerotic syndromes, including stable angina, cardiovascular diseases, stroke, and aortic and peripheral atherosclerosis, which subsequently result in intermittent claudication and abdominal aortic aneurysms.^29^

## 5 CONCLUSIONS

This study has found that longer puff times yield larger PM, whereas shorter puff times yield fine PM. For instance, at 15 s puff time, the mean particle aerodynamic diameters for MM and RR cigarettes, respectively, can be classified as coarse ≈ ${\mathrm{PM}}_{10}$. On the other hand, the mean particulate diameter at 2 s puff time for the standard cigarette falls under the ultrafine classification of $\leq {\mathrm{PM}}_{2.5}$, whereas at the same puff time, the mean particulate diameter for the local cigarette was approximately $\;{\mathrm{PM}}_{2.5}\;$, that is, considered fine PM. The consequences of inhaling fine or ultrafine particulates include cell mutation, cancer, emphysema and asthma, rheumatoid arthritis, oxidative stress, and coronary ailments. Accordingly, longer smoking times may benefit cigarette smokers because large particulates are less harmful than fine or ultrafine particulates that can penetrate deeper into the respiratory airway. Moreover, they have large surface areas that can induce severe inflammation and cell sensitization. Data from FTIR spectroscopy indicate that the PM in the two model cigarettes contains aromatic structures that feature the C=C bond characterized by an intense absorption band at δs (1600 cm^−1^). This implies the existence of harmful polycyclic aromatic hydrocarbons that may include naphthalene, anthracene, and benzo[a]pyrene. Bound long‐chain hydrocarbons onto cigarette PM are characterized by methyl and methylene group signatures that are lipophilic and can bind to the cell membrane, thus causing cell impairment and disease. Although CS was conducted for two model cigarettes, this study can be extended to any other form of cigarette. Moreover, this study emphasizes the need for comprehensive studies on real‐world CS conditions, taking into account cigarette smokers who use larger puff volumes.

## AUTHOR CONTRIBUTIONS

**Alfayo K. Maiyo:** Analysis; writing and editing. **Benjamin K. Korir:** Writing and editing. **Joshua K. Kibet:** Method development; editing; supervision. All authors have read and approved the manuscript.

## CONFLICT OF INTEREST

The authors have no competing interests.

## ETHICS STATEMENT

Not applicable.

## CONSENT FOR PUBLICATION

This article has the consent of all the authors.

## Data Availability

The data associated with the findings of this study are available from the corresponding author upon reasonable request.
